# Medical Comorbidities as the Independent Risk Factors of Severe Adenovirus Respiratory Tract Infection in Adults

**DOI:** 10.3390/microorganisms13071670

**Published:** 2025-07-16

**Authors:** Wang Chun Kwok, Isaac Sze Him Leung, James Chung Man Ho, David Chi Leung Lam, Mary Sau Man Ip, Shuk Man Ngai, Kelvin Kai Wang To, Desmond Yat Hin Yap

**Affiliations:** 1 Department of Medicine, The University of Hong Kong, Hong Kong, China; kwokwch@hku.hk (W.C.K.); jhocm@hku.hk (J.C.M.H.); dcllam@hku.hk (D.C.L.L.); msmip@hku.hk (M.S.M.I.); melanie1@hku.hk (S.M.N.); 2 Department of Statistics, The Chinese University of Hong Kong, Hong Kong, China; shleung@cuhk.edu.hk; 3 Department of Microbiology, School of Clinical Medicine, Li Ka Shing Faculty of Medicine, The University of Hong Kong, Hong Kong, China

**Keywords:** mortality, respiratory failure, secondary bacterial pneumonia, chronic kidney disease, dialysis

## Abstract

Adenovirus is an important respiratory virus that causes severe diseases in immunocompromised patients. Data on its impact in immunocompetent patients are relatively limited. We conducted a territory-wide retrospective study on adult patients hospitalized for respiratory tract infections caused by adenovirus or influenza viruses in Hong Kong between 1 January 2016 and 30 June 2023. Inpatient mortality, severe respiratory failure (SRF), secondary bacterial pneumonia and acute kidney injury (AKI) were compared. The risk factors for these outcomes in patients hospitalized for adenovirus respiratory tract infections were assessed. Overall, 41,206 and 528 patients were hospitalized for influenza and adenovirus respiratory tract infections, respectively. Patients with respiratory tract infections due to adenoviruses showed significantly higher risk of inpatient mortality, SRF, secondary bacterial pneumonia and AKI compared to seasonal influenza. Medical comorbidities including cardio-pulmonary diseases, end-stage kidney disease requiring dialysis, and a lower estimated glomerular filtration rate were robust independent risk factors for inpatient mortality and serious respiratory outcomes in adenovirus respiratory tract infections. Adults hospitalized for adenoviruses respiratory tract infections had a significantly higher risk of inpatient mortality and adverse outcomes than adults infected with seasonal influenza. Medical comorbidities are important risk factors for severe adenovirus infections in adult patients.

## 1. Introduction

Adenoviruses are non-enveloped, double-stranded DNA viruses consisting of over 50 serotypes [1]. Adenoviruses can cause respiratory and gastrointestinal tract infections. Adenovirus infection outbreaks can occur without any seasonality noted, especially in closed populations [2]. But these outbreaks may not be detected as the severity of the infections are mostly mild among immunocompetent subjects [3]. Severe adenoviruses infections were more common among immunocompromised patients, such as hematopoietic stem cell transplant recipients [4,5], solid organ transplant recipients [4,6,7,8,9], human immunodeficiency virus (HIV)-infected patients [4,10], and patients with congenital immunodeficiency [4].

Adenoviruses account for 1% to 7% of adult respiratory tract infections [11]. The respiratory tract manifestations include fever pharyngitis, tonsillitis, cough, and sore throat [12,13]. Pneumonia is relatively uncommon among immunocompetent adults [14,15,16,17].

In immunocompromised people, dissemination and/or severe respiratory failure can develop in up to 30% of patients [4,11,18,19] with mortality up to 50% [11,19,20]. Currently, the main treatment strategy for immunosuppressed hosts infected with adenovirus is mainly by reducing immunosuppressive treatment to restore the natural ability to combat infection [21]. No specific treatments have yet been approved for adenovirus infections, though cidofovir and other broad-spectrum antivirals such as ganciclovir and ribavirin have been reported to have some degree of efficacy at controlling adenovirus infections [22]. Although supplementing cidofovir treatment with intravenous immunoglobulin may exhibit some efficacy, it is not currently considered standard therapy [23]. With the advances in diagnostics and therapeutics, various potential therapeutic targets have been emerging which may bring about new hope in treating adenovirus infections. These include adenovirus-specific CD8^+^ T cells therapy [24] and engineered, antiviral monoclonal antibodies [25]. Repurposing approved compounds for the treatment of other pathologies can also be considered [22].

While current knowledge suggests that immunodeficiencies such as immunodeficiency syndrome and transplantation may be risk factors for severe adenovirus respiratory tract infections, there is a lack of evidence on other common medical comorbidities in relation to the risk of severe adenovirus respiratory tract infections. As it is one of the most commonly reported respiratory viral infections and new therapeutic strategies are under development and researched, it is necessary to evaluate the role of other medical comorbidities on the severity of adenovirus infections. This can help clinicians to decide on the target population most likely to benefit from these treatments. This may also help with vaccination strategies, as a vaccine against adenovirus is currently available [26]. As such, we conducted the current study to investigate common medical comorbidities, including chronic kidney disease, and the impact of patients hospitalized for adenovirus respiratory tract infections.

## 2. Materials and Methods

This was a territory-wide retrospective study to compare mortality and serious inpatient outcomes in adult patients hospitalized for adenoviruses and seasonal influenza respiratory tract infections. We also examined the risk factors for mortality and serious clinical outcomes in adult patients hospitalized for adenoviruses respiratory tract infections. Adult patients who were admitted to public hospitals in Hong Kong for adenoviruses infection and seasonal influenza respiratory tract infections between 1 January 2016 and 30 June 2023 were included. The patients were identified from the Hospital Authority’s (HA) Clinical Data Analysis and Reporting System (CDARS). CDARS is an electronic health record database managed by the HA. The HA is a public healthcare service provider covering more than 90% of Hong Kong’s population since 1993 [27,28,29]. Influenza and adenoviruses infections were detected by multiplex polymerase chain reaction (PCR) obtained from respiratory tract specimens. Subjects with dual infections with influenza and adenoviruses or co-infections with other respiratory viruses were excluded. The study was approved by the Institutional Review Board (IRB) of the University of Hong Kong and HA Hong Kong West Cluster (UW 24-137). Patient informed consent was waived in this retrospective study by the IRB as it is a retrospective study without active patient recruitment, and the data had already been de-identified. The study was conducted in compliance with the Declaration of Helsinki.

### 2.1. Outcome Measurements

The first main exposure of interest was adenoviruses and seasonal influenza infections. The main outcomes of interest were (1) death during hospitalization, (2) severe respiratory failure requiring invasive or non-invasive mechanical ventilation (SRF), (3) secondary bacterial pneumonia, and (4) acute kidney injury (AKI). AKI was defined according to the RIFLE criteria [30]. Secondary bacterial pneumonia was defined as the compatible radiologic changes on chest radiograph with supporting laboratory parameters (leukocytosis, neutrophilia) that necessitate systemic antibiotic treatment. First, we compared the incidence rates of the above outcomes between patients hospitalized for seasonal influenza and adenoviruses infections. Next, we examined the risk factors for the development of the above outcomes in patients with adenoviruses respiratory tract infections. The following covariates were assessed as potential risk factors associated with the outcomes: Age and Charlson comorbidity index (CCI) as continuous variables; gender; history of malignancy; underlying diabetes mellitus (DM), chronic airway diseases (asthma, chronic obstructive pulmonary disease [COPD], bronchiectasis), atherosclerotic cardio-/cerebro-vascular diseases (ischaemic heart disease, ischaemic stroke, peripheral vascular disease) and underlying kidney diseases as categorical variables. For underlying kidney diseases, patients were further divided into patients on dialysis (peritoneal dialysis [PD]/haemodialysis [HD] and chronic kidney disease (CKD) patients [defined as estimated glomerular filtration rate (eGFR) < 60 mL/min/1.73 m^2^) [31]; in addition, eGFR was assessed as a continuous variable at baseline.

### 2.2. Statistical Analysis

Descriptive tables were created to present the incidence rates of severe inpatient outcomes stratified by seasonal influenza and adenoviruses respiratory tract infections, with demographic and clinical data described as actual frequency or mean ± standard deviation (SD), or median [inter-quartile range (IQR)] where appropriate. Baseline demographic and clinical data were compared between the patients infected with seasonal influenza and adenoviruses respiratory tract infections using an independent *t*-test or Mann–Whitney U test where appropriate. To ensure a more meaningful comparison of patient outcomes, we also performed propensity score matching (PSM) to adjust for age, sex, ethnic group, history of malignancy, presence of DM, chronic airway diseases, atherosclerotic cardio-/cerebro-vascular diseases, ESKD on dialysis and CCI; with 1:1 matching and calliper of 0.2 times SD of the logit of the propensity score. Multiple imputations by chained equations were used to impute missing data for variables included in the adjusted analysis model.

To compare the risk of mortality and serious in-hospital complications between patients hospitalized with seasonal influenza and adenoviruses respiratory tract infections, we first performed univariate logistic regression analyses followed by multi-variable analysis. The risk factors for adverse clinical outcomes in patients hospitalized for adenoviruses respiratory tract infections were assessed first by univariate and then by multivariable analyses. The covariates adjusted in the multivariate analyses included age; sex; race, CCI; presence of DM, chronic airway diseases, atherosclerotic cardio-/cerebro-vascular diseases.

Data analyses were performed using R software version 4.0.3 (R Core Team, Vienna, Austria). For all statistical analyses, statistical significance was assessed at an α level of 0.05. STROBE and RECORD reporting guidelines were followed in the preparation of this report.

## 3. Results

### 3.1. Patients’ Characteristics

A total of 41,206 and 528 adult patients were admitted to public hospitals in Hong Kong for seasonal influenza and adenoviruses respiratory tract infections from 1 January 2016 to 30 June 2023 (Table 1). Patients with adenoviruses respiratory tract infections were younger, more often female, and had less DM, atherosclerotic cardio-/cerebro-vascular diseases than patients with seasonal influenza, but higher CCI (Table 1).

### 3.2. Severe In-Hospital Outcomes Among Influenza and Adenoviruses Patients

Adults hospitalized for adenoviruses infection had significantly higher rates of mortality (7.8% vs. 5.5%, *p* = 0.02), SRF (27.8% vs. 13.3%, *p* < 0.001), secondary bacterial pneumonia (51.7% vs. 39.5%, *p* < 0.001), and AKI (34.3% vs. 12.6%, *p* < 0.001) compared with seasonal influenza.

**Figure 1:**
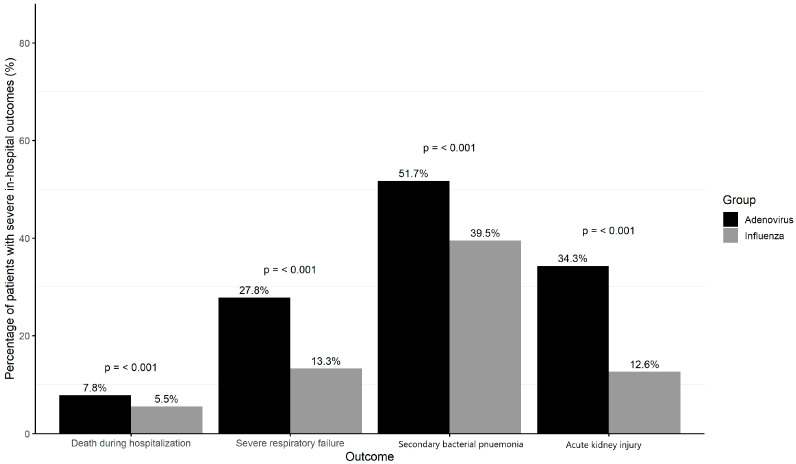
Severe in-hospital outcomes among influenza and adenovirus respiratory tract infections patients in whole cohort.

**Figure 2:**
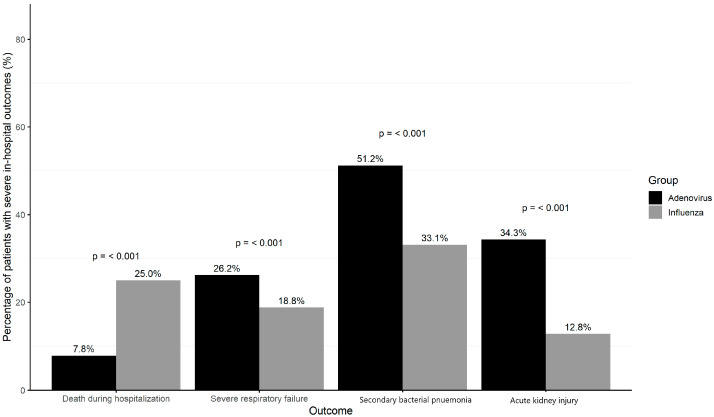
Severe in-hospital outcomes among influenza and adenovirus respiratory tract infections patients in propensity score-matched cohort.

Multivariable analyses demonstrated that the adenovirus infection was associated with a higher risk of death during hospitalization [adjusted odds ratio (aOR 4.63; 95% CI 3.21–6.69, *p* < 0.001), SRF (aOR 2.58; 95% CI 2.06–3.22 *p* < 0.001), secondary bacterial pneumonia (aOR 2.95; 95% CI 2.42–3.60, *p* < 0.001) and AKI (aOR 3.63; 95% CI 3.03–4.36, *p* < 0.001) than seasonal influenza (Figure 1). The results were largely consistent in the analyses using the 1:1 PSM cohort, except for risk of death during hospitalization, in which the risk was higher in patients with influenza infection (Figure 2).

### 3.3. Risk Factors for Severe In-Hospital Outcomes Among Adenovirus Patients

**Figure 3:**
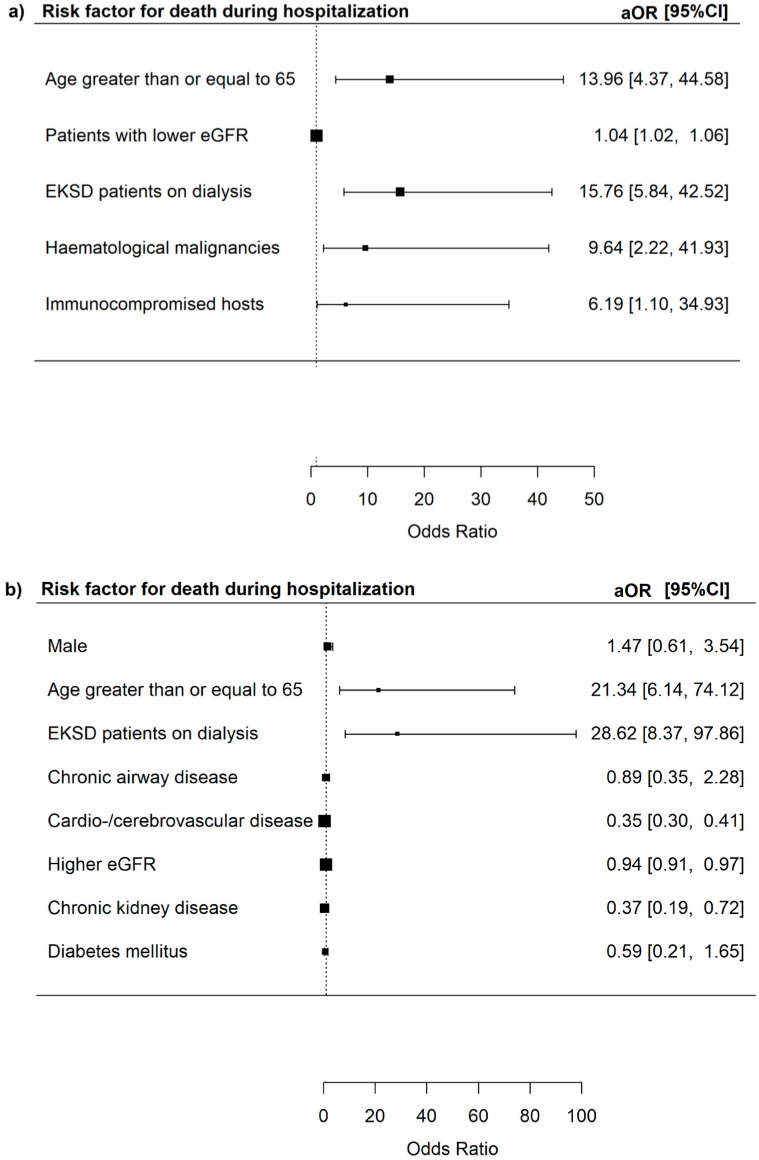
(**a**) Risk factors for death during hospitalization among all adenovirus respiratory tract infections patients. (**b**) Risk factors for death during hospitalization among adenovirus respiratory tract infections patients who did not have haematopoietic stem cell transplants, solid organ transplants, haematological malignancies, and HIV infections.

Next, we performed an analysis among adults hospitalized for adenovirus infection. The risk of mortality during hospitalization increased in patients aged ≥ 65 years (aOR 13.96, 95% CI = 4.37–44.58, *p* < 0.001), patients with lower eGFR (aOR 1.04, 95% CI = 1.02–1.06, *p* < 0.001), EKSD patients on dialysis (PD or HD) (aOR 15.76, 95% CI = 5.84–42.52, *p* < 0.001), patients with haematological malignancies (leukaemia/myeloma or lymphoma) (aOR 9.64, 95% CI = 2.22–41.93, *p* = 0.015) or immunocompromised hosts from transplantation, haematological malignancies, or HIV (aOR 6.19, 95% CI = 1.10–34.93, *p* = 0.039) (Figure 3).

**Figure 4:**
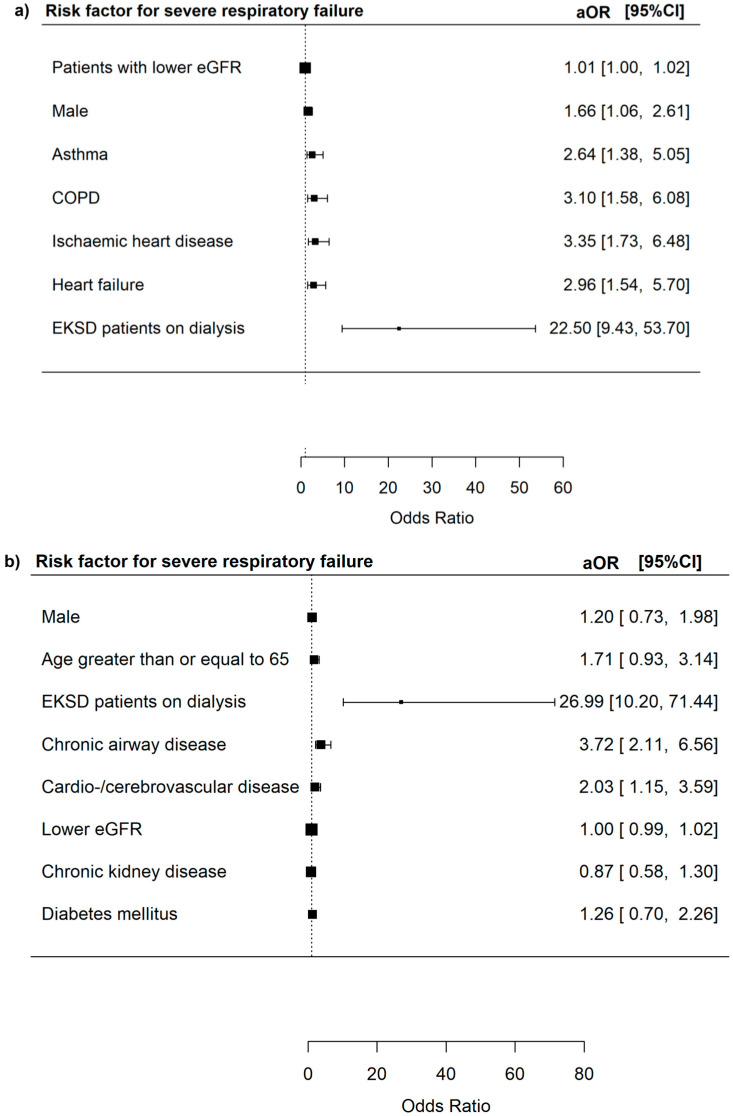
(**a**) Risk factors for severe respiratory failure among all adenovirus respiratory tract infections patients. (**b**) Risk factors for severe respiratory failure among adenovirus respiratory tract infections patients who did not have haematopoietic stem cell transplants, solid organ transplants, haematological malignancies, and HIV infections.

The risk of SRF was increased in patients with lower eGFR (aOR 1.01, 95% CI = 1.00–1.02, *p* = 0.012), male patients (aOR 1.66, 95% CI = 1.06–2.61 *p* = 0.027), patients with asthma (aOR 2.64 95% CI = 1.38–5.05, *p* < 0.001), COPD (aOR 3.10, 95% CI = 1.58–6.08, *p* = 0.001), ischaemic heart disease (aOR 3.35, 95% CI = 1.73–6.48, *p* < 0.001), and heart failure (aOR 2.96, 95% CI = 1.54–5.70, *p* = 0.001), and EKSD patients on dialysis (aOR 22.50, 95% CI = 9.43–53.70, *p* = < 0.001) (Figure 4).

**Figure 5:**
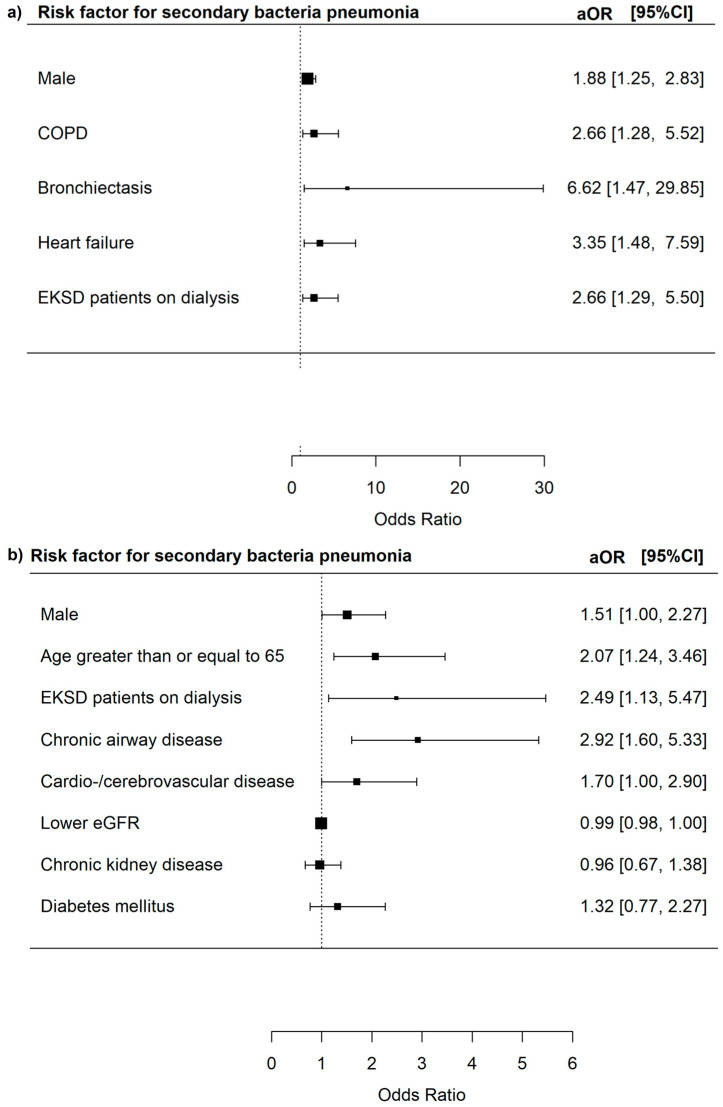
(**a**) Risk factors for secondary bacterial pneumonia among all adenovirus respiratory tract infections patients. (**b**) Risk factors for secondary bacterial pneumonia among adenovirus respiratory tract infections patients who did not have haematopoietic stem cell transplants, solid organ transplants, haematological malignancies, and HIV infections.

The risk of secondary bacteria pneumonia was increased in male patients (aOR 1.88, 95% CI = 1.25–2.83, *p* = 0.002), patients with COPD (aOR 2.66, 95% CI = 1.28–5.52, *p* = 0.009), bronchiectasis (aOR 6.62, 95% CI = 1.47–29.85, *p* = 0.014), and heart failure (aOR 3.35, 95% CI = 1.48–7.59, *p* = 0.004), and EKSD patients on dialysis (aOR 2.66, 95% CI = 1.29–5.50, *p* = 0.008) (Figure 5).

**Figure 6:**
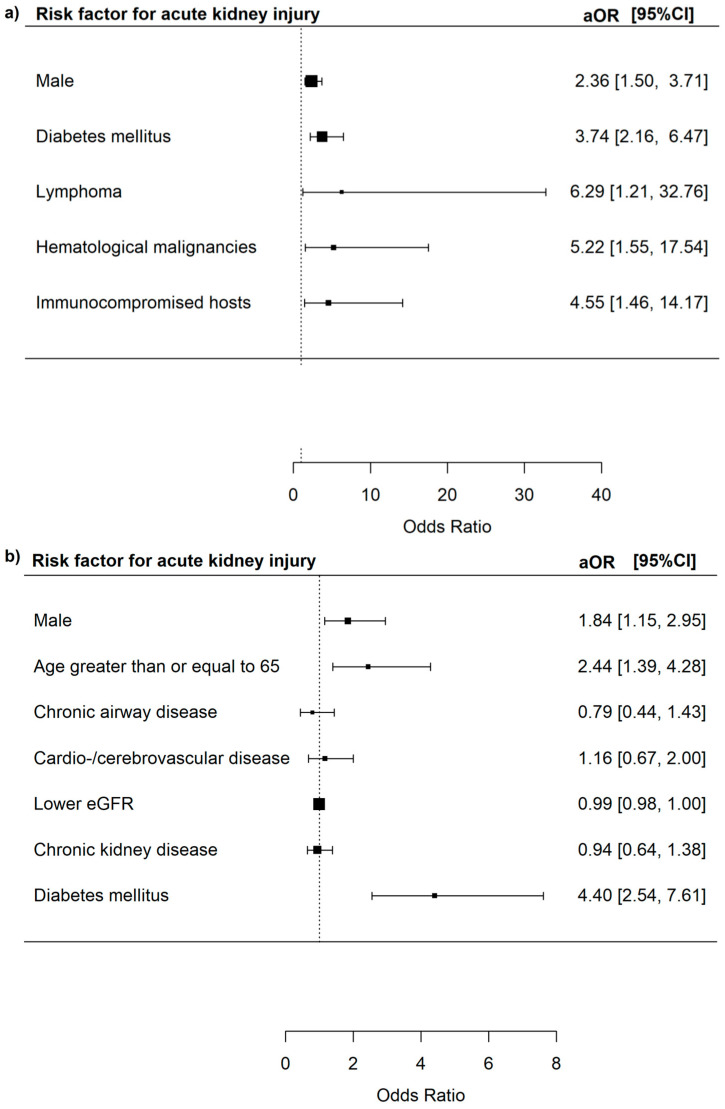
(**a**) Risk factors for acute kidney injury among all adenovirus respiratory tract infections patients. (**b**) Risk factors for acute kidney injury among adenovirus respiratory tract infections patients who did not have haematopoietic stem cell transplants, solid organ transplants, haematological malignancies, and HIV infections.

The risk of AKI was increased in male patients (aOR 2.36, 95% CI = 1.50–3.71, *p* < 0.001), those with DM (aOR 3.74, 95% CI = 2.16–6.47, *p* < 0.001), those with lymphoma (aOR 6.29, 95% CI = 1.21–32.76, *p* = 0.029), those with haematological malignancies (aOR 5.22, 95% CI = 1.55–17.54, *p* = 0.008) and those with immunocompromised hosts from transplantation, haematological malignancies, or HIV (aOR 4.55, 95% CI = 1.46–14.17, *p* = 0.009) (Figure 6).

### 3.4. Subgroup Analysis in Patients with Adenoviruses Infections

A subgroup analysis was performed for patients with adenoviruses infections who did not have haematopoietic stem cell transplants, solid organ transplants, haematological malignancies, and HIV infections, factors that have been shown to be associated with severe adenovirus infections. A total of 508 patients were included in the subgroup analysis.

The risk of death during hospitalization was increased in patients aged ≥65 years (aOR 21.34, 95% CI = 6.14–74.12, *p* < 0.001), patients with lower eGFR (aOR 1.06, 95% CI = 1.03–1.10, *p* < 0.001), and in EKSD patients on dialysis (aOR 28.62, 95% CI = 8.37–97.86, *p* < 0.001).

The risk of SRF was increased in patients with chronic airway diseases (aOR 3.72, 95% CI = 2.11–6.56, *p* < 0.001), atherosclerotic cardio-/cerebro-vascular diseases (aOR 2.03, 95% CI = 1.15–3.59, *p* = 0.015), and EKSD patients on dialysis (aOR 26.99, 95% CI = 10.20–71.44, *p* < 0.001).

The risk of secondary bacteria pneumonia was increased in male patients (aOR 1.51, 95% CI = 1.00–2.28, *p* = 0.048), those with chronic airway diseases (aOR 2.92, 95% CI = 1.60–5.33, *p* = 0.009), and EKSD patients on dialysis (aOR 2.49, 95% CI = 1.13–5.47, *p* = 0.023).

The risk of AKI was increased in male patients (aOR 1.84, 95% CI = 1.15–2.95, *p* = 0.044) and patients with DM (aOR 4.40, 95% CI = 2.54–7.61, *p* < 0.001).

## 4. Discussion

The results from this study suggest that adverse in-hospital outcomes were more commonly seen among adults hospitalized for adenoviruses respiratory tract infections than adults hospitalized for influenza. Medical comorbidities, especially cardiopulmonary diseases and dialysis-dependent ESKD, are risk factors for among adults hospitalized for severe adenovirus respiratory tract infections. Our findings shed light on vaccination strategies for adenoviruses infections.

Medical comorbidities have been identified as the risk factors for severe respiratory viral infections, such as influenza [32,33,34], respiratory syncytial virus (RSV) [35], and coronavirus disease 2019 (COVID-19) [36]. As such, vaccination strategies are also designed to target these risk groups. For adenovirus infection, immunocompromised state such as solid organ and haematopoietic stem cell transplantation were the most frequently reported risk factors for severe adenovirus infections. An American study conducted back in 2007 revealed that patients with chronic diseases were also at risk of severe adenovirus infection, but the authors did not stratify the patients into different underlying chronic diseases [37]. A more recent small-scale study also suggested that patients without severe immunosuppression are also at risk of severe adenovirus infection [38]. Our study provided detailed assessments of different medical comorbidities and their association with complications and mortality among adult patients without major immunosuppressed state hospitalized for adenoviruses infections. We were able to identify particular risk factors among these patients, namely chronic airway diseases, atherosclerotic cardio-/cerebro-vascular diseases and EKSD on dialysis. Of note, EKSD on dialysis was one of the risk factors associated with various respiratory complications and also with mortality. These findings should alert clinicians to the need to monitor these patients closely and to consider vaccination in this particular subgroup.

Defects in innate and adaptive immunity had been identified in CKD, making patients with CKD susceptible to infective complications [39,40,41]. A similar phenomenon has also been observed in COVID-19 [42,43,44] and influenza [45]. We were able to demonstrate the same phenomenon with adenoviruses. Not only was a lower baseline eGFR associated with various severe in-hospital outcomes, but patients with ESKD on dialysis also demonstrated to be at the highest risks of these severe in-hospital outcomes among all medical comorbidities. This finding suggested that adenovirus respiratory tract infections among patients on ddialysis should not be considered a mild form of respiratory tract infection. Adenoviruses are controlled by innate and adaptive immune responses involving various cytokines such as gamma interferon (IFN-γ), tumour necrosis factor (TNF), interleukin-1 (IL-1), IL-2, and macrophage inflammatory protein. The immunodeficiency state in CKD and ESKD could result in severe infections which can be related to ineffective viral clearance and viral persistence [46]. The close monitoring of the development of these complications is warranted as these patients are at risk of developing secondary bacterial pneumonia, SRF, and even death.

Advanced age, DM, atherosclerotic cardio-/cerebro-vascular diseases, and chronic airway diseases were also shown to be associated with severe in-hospital outcomes among patients hospitalized for adenovirus respiratory tract infections. This was also consistent with previous reports of chronic disease and severe adenovirus infections [29]. Similar phenomena have also been observed in influenza [32] and COVID-19 [36,47].

A vaccine is available for adenoviruses, but it is not widely prescribed. One of the populations that have used an adenovirus vaccine is the military population [26,48] to prevent outbreaks in military recruits. A live, oral, bivalent adenovirus vaccine was approved by the United States (US) Food and Drug Administration (FDA) that significantly reduced the incidence of respiratory infections by 50–60% and adenovirus-associated acute respiratory disease by 90% [49]. The reintroduction of new live vaccines in all military camps [50], led to a steady decline in the incidence of acute respiratory disease by 2014 [51,52]. Given the availability of an adenovirus vaccine that is not only clinically effective but also cost-effective among immunocompetent military recruits without major medical comorbidities [48,50,53], it is reasonable to consider adenovirus vaccination for high-risk groups that have major medical comorbidities (e.g., CKD and ESKD on dialysis) when available.

Our study has several limitations. First, some patient demographics, such as ethnicity, may be missing. We handled the missing data by multiple imputations. Second, we did not analyse secondary bacterial pneumonia in detail. Third, this was a study conducted in Hong Kong, and the majority of patients were Chinese. Nevertheless, in our multi-variable analysis and in the PSM cohort, we adjusted for most medical comorbidities and disease severity, and the results appeared to be consistent with our main analysis. We also performed subgroup analyses to ensure that our data was robust across different age groups. Furthermore, our data is derived from a territory-wide electronic health record system that captures comprehensive clinical information of all adults hospitalized for adenoviruses or seasonal influenza infection during the study period and is therefore a good representation of real-world data of this clinical entity.

## 5. Conclusions

Adults hospitalized for adenoviruses respiratory tract infections were associated with a significantly increased risk of in-patient mortality and adverse outcomes than those with seasonal influenza. Medical comorbidities, especially ESKD on dialysis, are important risk factors for severe adenoviruses infection among adult patients.

## Figures and Tables

**Table 1:** Baseline clinical characteristics for the full cohort and propensity score-matched (1:1) cohort.

		All	Whole Cohort		*p*-Value	ASD	Propensity Score-Matched Cohort		*p*-Value	ASD
			Influenza Virus	Adenovirus			Influenza Virus	Adenovirus		
Number of subjects (N)		41,975	41,447	528			485	485		
Age, years (Mean (SD))		67.20 (19.91)	67.37 (19.82)	54.30 (22.47)	<0.001 *	0.616	52.21 (20.95)	54.33 (22.58)	0.130	0.097 ^#^
Female (N, (%))		20,656 (49.2)	20,336 (49.1)	320 (60.6)	<0.001 *	0.233	293 (60.4)	289 (59.6)	0.844	0.017 ^#^
History of malignancies (N, (%))		2862 (6.8)	2794 (6.7)	68 (12.9)	<0.001 *	0.207	45 (9.3)	56 (11.5)	0.293	0.074 ^#^
Diabetes mellitus (N, (%))		10,223 (24.4)	10,133 (24.4)	90 (17.0)	<0.001 *	0.183	85 (17.5)	83 (17.1)	0.932	0.011 ^#^
Chronic airway diseases (N, (%))		6351 (15.1)	6268 (15.1)	83 (15.7)	0.750	0.017 ^#^	76 (15.7)	76 (15.7)	1.000	<0.001 ^#^
Cardiovascular diseases (N, (%))		14,063 (33.5)	13,932 (33.6)	131 (24.8)	<0.001 *	0.195	119 (24.5)	114 (23.5)	0.764	0.024 ^#^
CCI (Mean (SD))		3.74 (2.57)	3.75 (2.56)	3.09 (3.02)	<0.001 *	0.237	2.75 (2.72)	2.89 (2.91)	0.419	0.052 ^#^
CKD (N, (%))		7570 (18.0)	7470 (18.0)	100 (18.9)	0.626	0.024 ^#^	82 (16.9)	83 (17.1)	1.000	0.005 ^#^
Influenza vaccine (N, (%))		12,795 (30.5)	12,673 (30.6)	122 (23.1)	<0.001 *	0.169	109 (22.5)	112 (23.1)	0.878	0.015 ^#^
Pneumococcal conjugated vaccines (N, (%))		4787 (11.4)	4748 (11.5)	39 (7.4)	0.004 *	0.140	29 (6.0)	36 (7.4)	0.441	0.058 ^#^
Pneumococcal polysaccharide vaccine (N, (%))		1574 (3.7)	1557 (3.8)	17 (3.2)	0.596	0.029 ^#^	15 (3.1)	17 (3.5)	0.857	0.023 ^#^
ESKD on dialysis (N, (%))		1431 (3.4)	1378 (3.3)	44 (8.3)	<0.001 *	0.305	34 (7.0)	34 (7.0)	1.00	<0.001 ^#^
Leukaemia/Myeloma (N, (%))		222 (0.5%)	213 (0.5)	9 (1.7)	<0.001 *	0.164	5 (1.%)	6 (1.2)	0.762	0.064 ^#^
Lymphoma (N, (%))		219 (0.5%)	208 (0.5)	11 (2.1)	<0.001 *	0.218	11 (2.3)	8 (1.6)	0.487	0.064 ^#^
HIV infection (N, (%))		58 (0.1%)	54 (0.1)	4 (0.8)	<0.001 *	0.168	3 (0.6)	3 (0.6)	1.00	0.064 ^#^
Ethnicity (N, (%))					<0.001 *	0.270			1.00	0.015 ^#^
	Chinese	40,589 (96.7)	40,093 (96.7)	496 (93.9)			460 (94.8)	458 (94.4)		
	Northeast Asian	22 (0.1)	21 (0.1)	1 (0.2)			0 (0.0)	1 (0.2)		
	Southeast Asian	618 (1.5)	611 (1.5)	7 (1.3)			1 (0.2)	6 (1.2)		
	South Asian	512 (1.2)	510 (1.2)	2 (0.4)			1 (0.2)	2 (0.4)		
	Caucasian	17 (0.0)	17 (0.0)	0 (0.0)			0 (0.0)	0 (0.0)		
	Others	217 (0.5)	195 (0.5)	22 (4.2)			23 (4.7)	18 (3.7)		

SD = standard deviation; * = statistically significant; ASD = absolute standardized difference; # = good balance with ASD < 0.1; mmol = Millimoles per litre; ESKD = end-stage kidney disease; CKD = chronic kidney disease; CCI = Charlson comorbidity index.

## Data Availability

The original contributions presented in this study are included in the article. Further inquiries can be directed to the corresponding author.

## Abbreviations

The following abbreviations are used in this manuscript:

AKI	Acute Kidney Injury
CCI	Charlson Comorbidity Index
CDARS	Clinical Data Analysis and Reporting System
CKD	Chronic Kidney Disease
COPD	Chronic Obstructive Pulmonary Disease
DM	Diabetes Mellitus
eGFR	Estimated Glomerular Filtration Rate
ESKD	End-Stage Kidney Disease
HA	Hospital Authority
HIV	Human Immunodeficiency Virus
IQR	Inter-Quartile Range
IRB	Institutional Review Board
RIFLE	Risk, Injury, Failure, Loss of kidney function, and End-stage kidney disease
S.D.	Standard Deviation

## References

[1] Lynch J.P., Kajon A.E. (2016). Adenovirus: Epidemiology, Global Spread of Novel Serotypes, and Advances in Treatment and Prevention. Semin. Respir. Crit. Care Med..

[2] Kajon A.E., Lamson D.M., St George K. (2019). Emergence and re-emergence of respiratory adenoviruses in the United States. Curr. Opin. Virol..

[3] MacNeil K.M., Dodge M.J., Evans A.M., Tessier T.M., Weinberg J.B., Mymryk J.S. (2023). Adenoviruses in medicine: Innocuous pathogen, predator, or partner. Trends Mol. Med..

[4] Echavarria M. (2008). Adenoviruses in immunocompromised hosts. Clin. Microbiol. Rev..

[5] La Rosa A.M., Champlin R.E., Mirza N., Gajewski J., Giralt S., Rolston K.V., Raad I., Jacobson K., Kontoyiannis D., Elting L. (2001). Adenovirus infections in adult recipients of blood and marrow transplants. Clin. Infect. Dis..

[6] Humar A., Kumar D., Mazzulli T., Razonable R.R., Moussa G., Paya C.V., Covington E., Alecock E., Pescovitz M.D., Group P.V.S. (2005). A surveillance study of adenovirus infection in adult solid organ transplant recipients. Am. J. Transplant..

[7] McGrath D., Falagas M.E., Freeman R., Rohrer R., Fairchild R., Colbach C., Snydman D.R. (1998). Adenovirus infection in adult orthotopic liver transplant recipients: Incidence and clinical significance. J. Infect. Dis..

[8] Bridges N.D., Spray T.L., Collins M.H., Bowles N.E., Towbin J.A. (1998). Adenovirus infection in the lung results in graft failure after lung transplantation. J. Thorac. Cardiovasc. Surg..

[9] Ohori N.P., Michaels M.G., Jaffe R., Williams P., Yousem S.A. (1995). Adenovirus pneumonia in lung transplant recipients. Hum. Pathol..

[10] Hierholzer J.C. (1992). Adenoviruses in the immunocompromised host. Clin. Microbiol. Rev..

[11] Ison M.G. (2006). Adenovirus infections in transplant recipients. Clin. Infect. Dis..

[12] Ryan M.A., Gray G.C., Smith B., McKeehan J.A., Hawksworth A.W., Malasig M.D. (2002). Large epidemic of respiratory illness due to adenovirus types 7 and 3 in healthy young adults. Clin. Infect. Dis..

[13] Chang S.Y., Lee C.N., Lin P.H., Huang H.H., Chang L.Y., Ko W., Chang S.F., Lee P.I., Huang L.M., Kao C.L. (2008). A community-derived outbreak of adenovirus type 3 in children in Taiwan between 2004 and 2005. J. Med. Virol..

[14] Klinger J.R., Sanchez M.P., Curtin L.A., Durkin M., Matyas B. (1998). Multiple cases of life-threatening adenovirus pneumonia in a mental health care center. Am. J. Respir. Crit. Care Med..

[15] Cao B., Huang G.H., Pu Z.H., Qu J.X., Yu X.M., Zhu Z., Dong J.P., Gao Y., Zhang Y.X., Li X.H. (2014). Emergence of community-acquired adenovirus type 55 as a cause of community-onset pneumonia. Chest.

[16] Sanchez J.L., Binn L.N., Innis B.L., Reynolds R.D., Lee T., Mitchell-Raymundo F., Craig S.C., Marquez J.P., Shepherd G.A., Polyak C.S. (2001). Epidemic of adenovirus-induced respiratory illness among US military recruits: Epidemiologic and immunologic risk factors in healthy, young adults. J. Med. Virol..

[17] Kolavic-Gray S.A., Binn L.N., Sanchez J.L., Cersovsky S.B., Polyak C.S., Mitchell-Raymundo F., Asher L.V., Vaughn D.W., Feighner B.H., Innis B.L. (2002). Large epidemic of adenovirus type 4 infection among military trainees: Epidemiological, clinical, and laboratory studies. Clin. Infect. Dis..

[18] Symeonidis N., Jakubowski A., Pierre-Louis S., Jaffe D., Pamer E., Sepkowitz K., O’Reilly R.J., Papanicolaou G.A. (2007). Invasive adenoviral infections in T-cell-depleted allogeneic hematopoietic stem cell transplantation: High mortality in the era of cidofovir. Transpl. Infect. Dis..

[19] Kim Y.J., Boeckh M., Englund J.A. (2007). Community respiratory virus infections in immunocompromised patients: Hematopoietic stem cell and solid organ transplant recipients, and individuals with human immunodeficiency virus infection. Semin. Respir. Crit. Care Med..

[20] Hakim F.A., Tleyjeh I.M. (2008). Severe adenovirus pneumonia in immunocompetent adults: A case report and review of the literature. Eur. J. Clin. Microbiol. Infect. Dis..

[21] Al-Heeti O.M., Cathro H.P., Ison M.G. (2022). Adenovirus Infection and Transplantation. Transplantation.

[22] Dodge M.J., MacNeil K.M., Tessier T.M., Weinberg J.B., Mymryk J.S. (2021). Emerging antiviral therapeutics for human adenovirus infection: Recent developments and novel strategies. Antivir. Res..

[23] Haq A., Gregston A., Elwir S., Spak C.W. (2022). Treatment of Viral Hepatitis Due to Adenovirus in a Liver Transplantation Recipient: The Clinical Use of Cidofovir and Intravenous Immunoglobulin. Liver Transpl..

[24] Dailey Garnes N.J.M., Ragoonanan D., Aboulhosn A. (2019). Adenovirus infection and disease in recipients of hematopoietic cell transplantation. Curr. Opin. Infect. Dis..

[25] Foss S., Jonsson A., Bottermann M., Watkinson R., Lode H.E., McAdam M.B., Michaelsen T.E., Sandlie I., James L.C., Andersen J.T. (2022). Potent TRIM21 and complement-dependent intracellular antiviral immunity requires the IgG3 hinge. Sci. Immunol..

[26] Iskander J., Blanchet S., Springer C., Rockwell P., Thomas D., Pillai S. (2023). Enhanced Adenovirus Vaccine Safety Surveillance in Military Setting, United States. Emerg. Infect. Dis..

[27] Kwok W.C., Tam T.C.C., Sing C.W., Chan E.W.Y., Cheung C.L. (2023). Validation of diagnostic coding for bronchiectasis in an electronic health record system in Hong Kong. Pharmacoepidemiol. Drug Saf..

[28] Kwok W.C., Tam T.C.C., Sing C.W., Chan E.W.Y., Cheung C.L. (2023). Validation of Diagnostic Coding for Asthma in an Electronic Health Record System in Hong Kong. J. Asthma Allergy.

[29] Gao L., Leung M.T.Y., Li X., Chui C.S.L., Wong R.S.M., Au Yeung S.L., Chan E.W.W., Chan A.Y.L., Chan E.W., Wong W.H.S. (2021). Linking cohort-based data with electronic health records: A proof-of-concept methodological study in Hong Kong. BMJ Open.

[30] Bellomo R., Ronco C., Kellum J.A., Mehta R.L., Palevsky P., Acute Dialysis Quality Initiative w. (2004). Acute renal failure - definition, outcome measures, animal models, fluid therapy and information technology needs: The Second International Consensus Conference of the Acute Dialysis Quality Initiative (ADQI) Group. Crit. Care.

[31] Levey A.S., Eckardt K.U., Tsukamoto Y., Levin A., Coresh J., Rossert J., De Zeeuw D., Hostetter T.H., Lameire N., Eknoyan G. (2005). Definition and classification of chronic kidney disease: A position statement from Kidney Disease: Improving Global Outcomes (KDIGO). Kidney Int..

[32] Mertz D., Kim T.H., Johnstone J., Lam P.P., Science M., Kuster S.P., Fadel S.A., Tran D., Fernandez E., Bhatnagar N. (2013). Populations at risk for severe or complicated influenza illness: Systematic review and meta-analysis. BMJ.

[33] Cocoros N.M., Lash T.L., DeMaria A., Klompas M. (2014). Obesity as a risk factor for severe influenza-like illness. Influenza Other Respir. Viruses.

[34] Dicembrini I., Silverii G.A., Clerico A., Fornengo R., Gabutti G., Sordi V., Tafuri S., Peruzzi O., Mannucci E. (2023). Influenza: Diabetes as a risk factor for severe related-outcomes and the effectiveness of vaccination in diabetic population. A meta-analysis of observational studies. Nutr. Metab. Cardiovasc. Dis..

[35] Andreas A., Doris L., Frank K., Michael K. (2023). Corrigendum to Focusing on severe infections with the Respiratory Syncytial Virus (RSV) in adults: Risk factors, symptomatology and clinical course compared to influenza A/B and the original SARS-CoV-2 strain: Journal of Clinical Virology, 161 (2023), 105399. J. Clin. Virol..

[36] Gao Y.D., Ding M., Dong X., Zhang J.J., Kursat Azkur A., Azkur D., Gan H., Sun Y.L., Fu W., Li W. (2021). Risk factors for severe and critically ill COVID-19 patients: A review. Allergy.

[37] Gray G.C., McCarthy T., Lebeck M.G., Schnurr D.P., Russell K.L., Kajon A.E., Landry M.L., Leland D.S., Storch G.A., Ginocchio C.C. (2007). Genotype prevalence and risk factors for severe clinical adenovirus infection, United States 2004–2006. Clin. Infect. Dis..

[38] Cederwall S., Pahlman L.I. (2020). Respiratory adenovirus infections in immunocompetent and immunocompromised adult patients. Epidemiol. Infect..

[39] Yap D.Y., Fong C.H., Zhang X., Ip J.D., Chan W.M., Chu A.W., Chen L.L., Zhao Y., Chan B.P., Luk K.S. (2023). Humoral and cellular immunity against different SARS-CoV-2 variants in patients with chronic kidney disease. Sci. Rep..

[40] Smits P.D., Gratzl S., Simonov M., Nachimuthu S.K., Goodwin Cartwright B.M., Wang M.D., Baker C., Rodriguez P., Bogiages M., Althouse B.M. (2023). Risk of COVID-19 breakthrough infection and hospitalization in individuals with comorbidities. Vaccine.

[41] Ma B.M., Yap D.Y.H., Yip T.P.S., Hung I.F.N., Tang S.C.W., Chan T.M. (2021). Vaccination in patients with chronic kidney disease-Review of current recommendations and recent advances. Nephrology.

[42] Xu C., Zhang T., Zhu N., Han M. (2021). Characteristics of COVID-19 patients with preexisting CKD history. Int. Urol. Nephrol..

[43] Pio-Abreu A., do Nascimento M.M., Vieira M.A., de Menezes Neves P.D.M., Lugon J.R., Sesso R. (2020). High mortality of CKD patients on hemodialysis with COVID-19 in Brazil. J. Nephrol..

[44] Akchurin O., Meza K., Biswas S., Greenbaum M., Licona-Freudenstein A.P., Goyal P., Choi J.J., Choi M.E. (2021). COVID-19 in Patients with CKD in New York City. Kidney360.

[45] Kang S.H., Cheong H.J., Song J.Y., Noh J.Y., Jeon J.H., Choi M.J., Lee J., Seo Y.B., Lee J.S., Wie S.H. (2016). Analysis of Risk Factors for Severe Acute Respiratory Infection and Pneumonia and among Adult Patients with Acute Respiratory Illness during 2011-2014 Influenza Seasons in Korea. Infect. Chemother..

[46] Lion T. (2014). Adenovirus infections in immunocompetent and immunocompromised patients. Clin. Microbiol. Rev..

[47] Zhang J.J., Dong X., Liu G.H., Gao Y.D. (2023). Risk and Protective Factors for COVID-19 Morbidity, Severity, and Mortality. Clin. Rev. Allergy Immunol..

[48] Eom J., Kim Y., Kim D., Lee E., Kwon S.H., Jo M.W., Jung J., Park H., Park B. (2024). Cost-benefit analysis of human adenovirus vaccine development in a Korean military setting. Vaccine.

[49] Dudding B.A., Top F.H., Winter P.E., Buescher E.L., Lamson T.H., Leibovitz A. (1973). Acute respiratory disease in military trainees: The adenovirus surveillance program, 1966–1971. Am. J. Epidemiol..

[50] Radin J.M., Hawksworth A.W., Blair P.J., Faix D.J., Raman R., Russell K.L., Gray G.C. (2014). Dramatic decline of respiratory illness among US military recruits after the renewed use of adenovirus vaccines. Clin. Infect. Dis..

[51] Hoke C.H., Snyder C.E. (2013). History of the restoration of adenovirus type 4 and type 7 vaccine, live oral (Adenovirus Vaccine) in the context of the Department of Defense acquisition system. Vaccine.

[52] Clemmons N.S., McCormic Z.D., Gaydos J.C., Hawksworth A.W., Jordan N.N. (2017). Acute Respiratory Disease in US Army Trainees 3 Years after Reintroduction of Adenovirus Vaccine. Emerg. Infect. Dis..

[53] Hyer R.N., Howell M.R., Ryan M.A., Gaydos J.C. (2000). Cost-effectiveness analysis of reacquiring and using adenovirus types 4 and 7 vaccines in naval recruits. Am. J. Trop. Med. Hyg..

